# Isolation of *Mycobacterium avium* from Potable Water in Homes and Institutions of Patients with HIV Infection in Finland and the United States

**DOI:** 10.1155/2015/713845

**Published:** 2015-06-09

**Authors:** Matti Ristola, Robert D. Arbeit, C. Fordham von Reyn, C. Robert Horsburgh

**Affiliations:** ^1^Division of Infectious Diseases, University of Helsinki and Helsinki University Hospital, HUS, PL 348, 00029 Helsinki, Finland; ^2^Tufts University School of Medicine, 136 Harrison Avenue, Boston, MA 02111, USA; ^3^Geisel School of Medicine at Dartmouth, DHMC, One Medical Center Drive, Lebanon, NH 03756, USA; ^4^Center for Global Health and Development, Boston University School of Public Health, 801 Massachusetts Avenue, Room 389, Boston, MA 02118, USA

## Abstract

Symptomatic disease by nontuberculous mycobacteria has been linked to potable water from institutional and domestic potable water systems. Potable water samples were collected from homes and institutions of patients with AIDS. Colonization of potable water with nontuberculous mycobacteria was demonstrated in 230 (15%) of 1489 samples collected from domestic and institutional water systems of patients with HIV infection in the United States and Finland. *Mycobacterium avium* was the most common species and colonization was favored at temperatures of 40–50°C in recirculating hot water systems. Such systems are a plausible source of human infection and disease.

## 1. Introduction

Nontuberculous mycobacteria, including organisms of the* Mycobacterium avium* complex (MAC), may cause asymptomatic infections identified by positive skin test reactions and also symptomatic disease including lymphadenitis, pulmonary infection, and disseminated disease identified by positive bacterial cultures [1]. MAC and other nontuberculous mycobacteria (NTM) are environmental organisms that can be isolated from diverse soil and water sources [2]. Symptomatic disease has been linked to exposure to potable water including both institutional and domestic potable water systems [3, 4]. Asymptomatic infection has been linked to occupational soil exposure [5].

## 2. Materials and Methods

In an international epidemiologic study we obtained potable water samples from homes and institutions of patients with AIDS and followed up patients for the development of disseminated MAC [6]. We have previously reported on the 40 cases of disseminated MAC in patients from the United States and Finland including 7 cases that could be linked by molecular typing methods to institutional hot water colonized with the identical strain [3] and 2 clusters of identical patient isolates in Finland [7] and one case that could be linked to a domestic sample [6]. In the present report we present the results of cultures for MAC and other NTM from potable domestic and institutional sources to which the patients were exposed and identify factors favoring colonization of water systems.

## 3. Results

We collected 1489 water samples from domestic and institutional (workplaces, hospitals) sources during a multicenter study of disseminated* Mycobacterium avium* infection among HIV-infected patients in the United States (Atlanta, Boston and New Hampshire) and in Finland [6, 8]. All patients gave at least 2 potable water samples from their residence. In addition, up to 4 water samples were obtained from other sites where patients had regular contact with water. The maximum temperature of hot water, type of building, and type of water system were recorded. Laboratory methods have been described previously [8]. The study was approved by institutional review boards at all sites [8].

**Figure 1:**
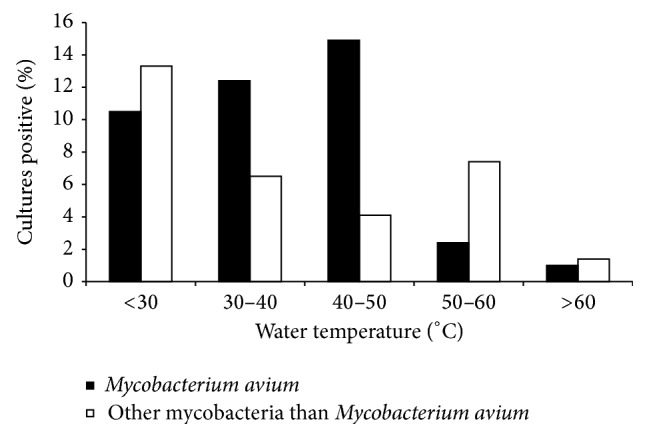
Colonization rates by water temperature.

**Figure 2:**
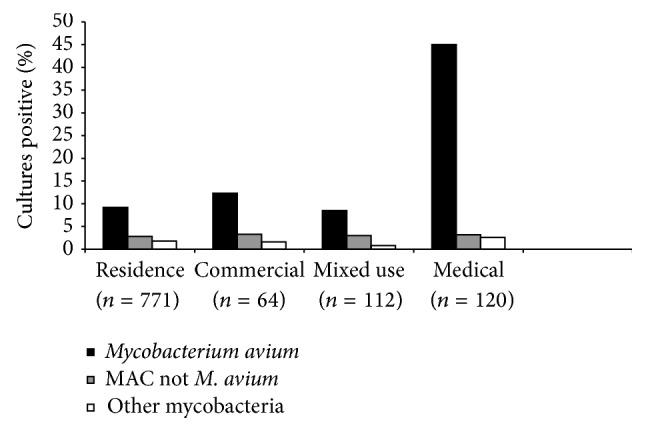
Colonization rates by building use.

A total of 230 (15%) samples were positive for NTM including 144 (63%) for* M. avium*; 32 (14%) for* M. avium* complex (MAC), not* M. avium*; and 54 (23%) for other NTM. Isolation of* M. avium* was highest at temperatures of 40–50°C and lowest at temperatures > 60°C. In contrast, isolation of any NTM was highest at temperatures < 30°C and fell progressively with increasing temperatures (Figure 1).* M. avium* grew from 65 (32%) of 204 samples from recirculating hot water systems (*n* = 204) compared to 10 (2%) of 619 samples from nonrecirculating water systems (*p* < 0.0001). Forty-five percent of samples from medical institutions were positive for* M. avium* compared to 9 to 12% of samples from residential or other types of buildings (Figure 2).

## 4. Discussion

Infection with NTM can rarely lead to symptomatic disease: cervical adenitis in children, pulmonary disease in adults with and without underlying lung pathology, and disseminated disease in patients with HIV infection [1]. However, skin test studies indicate that asymptomatic infection with NTM such as* M. avium* is common in healthy persons throughout the world [9]. These asymptomatic infections have both favorable and unfavorable implications for human health: on the one hand they may confer protection against disease due to* M. tuberculosis* and on the other may interfere with immune responses to live mycobacterial vaccines such as BCG [2].

The diverse sources of these infections in healthy persons are not well understood. The present study indicates that* M. avium* colonization of potable hot water systems is common and represents a potential source of both asymptomatic infection and symptomatic disease. Water temperatures 40–50°C have the highest colonization rates and rates are low at >60°C. Further, the recirculating hot water systems generally used in institutions have the potential for persistence of NTM in biofilms and are more likely to be colonized than the one way direct distribution systems generally used in single family homes.

Since exposure to hot water sources is almost universal and since colonization of water systems is common, it would be difficult to design a conventional epidemiologic study to confirm water exposure as a transmission risk. We did not link isolates from the present study with human disease. However, in previous studies we have used molecular epidemiology to confirm transmission from hospital hot water to patients with HIV infection [3] and numerous epidemiologic studies have linked nosocomial NTM infection with colonized water sources. Soil is another potential source of infection in healthy subjects as demonstrated by the universal colonization of soil with NTM in Finland [10] and supported by a skin test study of healthy persons in Belle Glade Florida [5].

Collectively these emerging data suggest a hypothesis for acquisition of asymptomatic NTM infection in the northern hemisphere. In the United States where infection due to* M. tuberculosis* is uncommon, antibody to the common mycobacterial antigen lipoarabinnomanin rises with age and supports widespread age-related acquisition of asymptomatic NTM infection [11]. Symptomatic cervical adenitis due to NTM is rare before age 1 and occurs principally during ages 1–5 years, suggesting that not only symptomatic disease but possibly asymptomatic infection might result from initial exposure to NTM in soil (or water) during the ages when teeth are erupting and hygiene is not fully developed. Among older children and adults the frequent colonization of water documented in the present study could provide the source of additional asymptomatic infections.

Recent studies and our own investigations belie the general belief that infections with NTM are more common in the southern than in the northern hemisphere [2]. We have shown previously that skin test reactivity to* Mycobacterium avium* complex (MAC), indicating prior asymptomatic infection, can be demonstrated in 35–48% of healthy adults in the northern and southern United States, respectively, and among a substantial proportion of healthy adults in Finland [8, 12]. Further, overt disease due to NTM is uncommon in the southern hemisphere [13] and asymptomatic MAC infection of healthy persons detected by identical skin testing methods is no more common in Africa than in the United States [8]. Although exposure to soil might be more common among both children and adults in resource-limited countries, access to NTM-colonized hot water systems might be expected to be less common in these same regions.

## 5. Conclusions

We have shown that colonization of hot water systems with NTM, especially* M. avium*, is common in the United States and Finland. Colonization by* M. avium* is favored at temperatures of 40–50°C and in recirculating systems. Hot water sources are a potential source for the many asymptomatic infections with NTM that can be documented by skin testing among persons living in the northern hemisphere.
